# Effect of peptide-conjugated nanoparticles on cell lines

**DOI:** 10.1007/s40204-019-0106-9

**Published:** 2019-01-19

**Authors:** Kangkana Banerjee, V. Ravishankar Rai, M. Umashankar

**Affiliations:** 1 Department of Studies in Microbiology, University of Mysore, Manasagangothri, Mysore, Karnataka 570006 India; 2 Department of Chemistry, KSOU, Manasagangothri, Mysore, Karnataka 570006 India

**Keywords:** Gold nanoparticles (AuNPs), Peptides, Conjugation, Cancer cell lines, Apoptosis

## Abstract

Metal nanoparticles are widely used for the delivery and targeting of pharmaceutical, therapeutic and diagnostic agents in cancer therapy in recent years. The multifunctional nanoparticles constructed currently are supposed to show superior effects on cancer cells. This study was conducted to observe the difference between the effect of a biologically important peptide, silver (AgNPs) and gold (AuNPs) nanoparticles and their conjugates on two different cancer cells. Peptide (Boc-L-^D^P-L-OMe) was acquired from different sources and subjected to conjugation with biosynthesized gold and silver nanoparticles under standard conditions. These conjugates were tested against the colon cancer (HT-29) and breast cancer (MDA MB-231) cell lines. The results clearly depicted the improved activity of nanoparticles in the form of conjugates. Fluorescent dye microscopy and DNA fragmentation assay substantiate the fact that the conjugated nanoparticles cause higher level of disintegration of DNA in cells that consecutively damages and causes apoptosis due to lethality.

## Introduction

The existing therapeutics obtained from nanoparticles is chiefly site specific or targeted in nature. Presently, enormous advances in cancer healing and various significant applications associated with drug therapy in medical science field are being observed (Shrivastava et al. 2007). The capability of nanoparticles to perform on site-specific areas has been a vital feature in the functionalization studies in nano-biotechnology, which, thus, avoids disturbing healthy tissues and unnecessary side effects of such drugs (Gindy and Prud’homme 2009). The importance of harsh chemical and physical therapy in cancer cure is one more drawback of usual medical remedies. The method, in which the drugs change the healthy tissue in our body alongside the cancer cells and tissues, is caused by their nonspecificity of therapeutic drugs. Heath and Davis stated, the actuality that drugs can be merely administered in later stages in cancer growth and metastatic ailment, adds a superior possibility to death (Heath and Davis 2008). Thus, current management methods in nanotechnology recommend better therapy due to their tiny size which, when coupled to larger bioactive particles, offers effective outcome compared to unaided biological molecules and vice versa.

Silver and gold nanoparticles have traditionally been believed to be superior compounds for a range of therapies and recently, they are functionalized as drug-delivery vehicles in broad range of analyses. Among all these systems, one of the main elements is developing methodologies to properly manufacture nanoparticles to have the best effects. Targeted drug delivery assists improved concentration of drugs at the location of disease which consecutively lessen side effects, enhance efficacy, or achieve some of both sometimes (Hambley and Hait 2009, Ruoslahti et al. 2017). Also, peptide–NP conjugates develop the stability of the nanoparticles during their interaction with the cells and their organelles.

This study principally aims to conclude the effect of these nanoparticles hybridized to a biologically essential peptide on colon cancer cells. Additionally, we attempt to comprehend the apoptotic approach of the peptide-conjugated nanoparticles in preventing cancer-cells growth. The results obtained were systematically compared with mouse embryo fibroblast (NIH3T3) cells.

## Experimental

### Peptide synthesis

Peptides P1 (Boc-L-^D^P-L-OMe), P2 ((U^B^F)_4_-OMe), P3 (Boc-P-A-OH), P4 (Boc-P-U-OH), P5 (Boc-βGly-βGly-OMe) were synthesized by conventional solution phase chemistry using a racemization-free fragment condensation strategy as explained by Rai et al. (2006). The final peptides were characterized by the mass spectrometer research group.

### Screening of cytotoxic activity of peptides

Above-mentioned peptides (P1, P2, P3, P4, and P5) obtained from other sources were screened for cytotoxicity by MTT assay on NIH3T3 (Mouse embryo fibroblast) cells (normal) and HT-29 (Colon carcinoma) cells (cancerous) by MTT assay. Different concentrations of the peptides (1 μL, 3 μL, 5 L, 10 μL, 20 μL) were used to treat the cancerous and normal cells consecutively (Inkielewicz-Stepniak et al. 2014). The percentage inhibition was calculated.

### Peptide–NP conjugate synthesis

Peptide P1 was modified by replacing cysteine for the easy assembly of the nanoparticles. Particles were conjugated to peptide P1 (Boc-L-^D^P-L-OMe) by standardized procedures. Briefly, nanoparticles (silver and gold) and P1 were initially dissolved in 1% DMSO and then mixed at the desired ratio and allowed to self-assemble for 30 min before use. The mixture was then sonicated for 3–5 min for uniform combining.

### Characterization of conjugates

The conjugation efficiency was monitored by UV–Vis spectroscopy by scanning the resultant solution in the range of 200–800 nm for P1–silver NP and P1–gold NP conjugates consecutively. Particles were purified of excess P1 by centrifugation and washing. The resultant solution was characterized by dynamic light scattering (DLS) to obtain the resultant particle size and the polydispersity index (PDI).

### MTT assay

Cell lines were acquired from NCCS—National Centre for Cell Science, Pune, India. The HT-29 (Colon carcinoma) and MDA MB-231 (Breast cancer) cells were plated into 25-mm^2^ T-flasks after thawing them at 37 °C in a water bath for few minutes. The thawed cells were transferred in a centrifuge tube and centrifuged for 3 min at 2000 rpm/min. The cells were, thus, collected discarding the supernatant and mixed well in Dulbecco’s modified Eagle’s medium (DMEM) solution with 12% fetal bovine serum, and antibiotics (streptopenicillin and amphotericin B solution, Sigma). Later, they were plated in 25 mm^2^ in the same DMEM solution, individually. The T-flasks containing the cells were incubated for 24 h in humidified environment with 5% CO_2_. These cells after 24 h were washed with phosphate buffer saline (PBS). The medium was regularly changed for the cells to reach 50% confluence (Hajiaghaalipour et al 2015). To check the viability of cells, MTT assay was carried out and 96-well plates were used to seed the cell lines of interest at 5000 cells/well and incubated overnight at 37 °C in an incubator with 5% CO_2_. After incubation, the cells were treated with different concentrations (1, 5, 10 μg/mL) of P1, AgNPs, AuNPs, P1–AgNP and P1–AuNP conjugates. The plates along with the treated cells were again incubated for 24 h at the same condition. The stored stock of MTT solution was added to the control and treated cell line samples, and again incubated at 37 °C for 2–3 h. After incubation, the media were carefully removed from the wells and mixed with solubilizing buffer. A purple color solution was visible at this stage. The control solution (without incorporation of nanoparticles/pure incubated cells) and treated samples were again incubated for an hour at 37 °C to ensure that the formazan precipitate was dissolved. The measurement of MTT assay was analyzed with Elisa Reader (Thermo Scientific, Multiskan Spectrum) at 570 nm (Inkielewicz-Stepniak et al 2014). Percentage inhibition of cells was calculated according to the following equation:

$${\text{Cytotoxicity}}\;(\% ) = ({\text{experimental}}\;{\text{absorbance}}\; 5 70\;{\text{nm}}\;{\text{of}}\;{\text{exposed}}\;{\text{cells}}/{\text{absorbance}}\; 5 70\;{\text{nm}}\;{\text{of}}\;{\text{unexposed}}\;{\text{cells}}) \times 100.$$

### Fluorescent imaging

#### Propidium iodide/acridine orange staining for dead/live cells

HT-29 and MDA MB-231 cells were plated at a density of 1 × 10^6^ cells/mL in 6-well tissue culture plates. They were treated with 1 µg/mL each of AgNP, AuNP, P1, P1–AgNP and P1–AuNP conjugated samples and were incubated in at 37 °C in 5% CO_2_ for 24 h. The cells were then harvested and washed twice using PBS after centrifugation at 2000 rpm for 5 min. An equal volume (10 µg/mL) of fluorescent dyes, propidium iodide (PI)/acridine orange (AO) was added to the cellular pellet. These freshly stained cells were observed under an inverted fluorescent microscope within 30 min (Banerjee and Ravishankar 2017).

### DAPI staining

DAPI (4’,6-diamidino-2-phenylindole) is a fluorescent compound that binds to the adenine–thymine-rich DNA and therefore, it stains the live cells. Since NIH3T3 cells do not show much change due to treatment with AgNP, AuNP, P1, P1–AgNP and P1–AuNP conjugated samples, we continued our study with cancer cell lines (HT 29). The HT-29 and MDA MB-231 cells are treated with 1 μg/mL each of the above-mentioned samples according to previous assay results and stained with DAPI, and observed under fluorescence microscope (Axio Imager A2, Carl Zeiss, Germany) (Hashim et al 2013).

### DNA fragmentation assay

HT-29 and MDA MB-231 cell lines were plated in 24 flat-well plates at 50,000 cells/well concentration in DMEM and incubated for 24 h. The cells were, thus, treated with 1 µg/mL concentration each of P1, AgNP, AuNP, P1-AgNP and P1-AuNP conjugates. After 24–30-h incubation, cells were harvested and re-suspended with 1-mL PBS, washed by centrifugation. The supernatant was then discarded and 500-µL lysis buffer is added (40 mL of 0.5 M EDTA, 5 mL of 1 M Tris-Cl buffer pH 8.0, 5 mL of 100% Triton X-100, 50 mL of H_2_O), incubated for 5 min at 65 °C. A mixture of chloroform–isoamyl alcohol (700 µL) was added after cooling down the solution to room temperature. The mixture was centrifuged at 12,000 rpm for 5 min. The aquatic phase obtained was then transferred to fresh Eppendorf to which equal volume of cold isopropanol was added. This solution was further centrifuged at 12,000 rpm for 5 min. The supernatant was discarded and pellets were dried before dissolving the dried DNA in 50 µL of distilled water. The DNA was resolved in 0.8% agarose gel (Balaji and Gothandam 2016).

### Statistical analysis

All experiments were done in triplicates and the results were represented as mean ± SEM. The experimental data were read by two-way analysis of variance (ANNOVA). Differences were considered significant at *p* < 0.05. The IC-50 value was calculated using Microsoft Excel 2007 software (logarithmic transformation of *X* values and nonlinear regression sigmoidal dose–response analysis with variable slope—with bottom and top constraints set at 0 and 100, resp.).

## Results and discussion

### Cytotoxicity of peptides

**Fig. 1:**
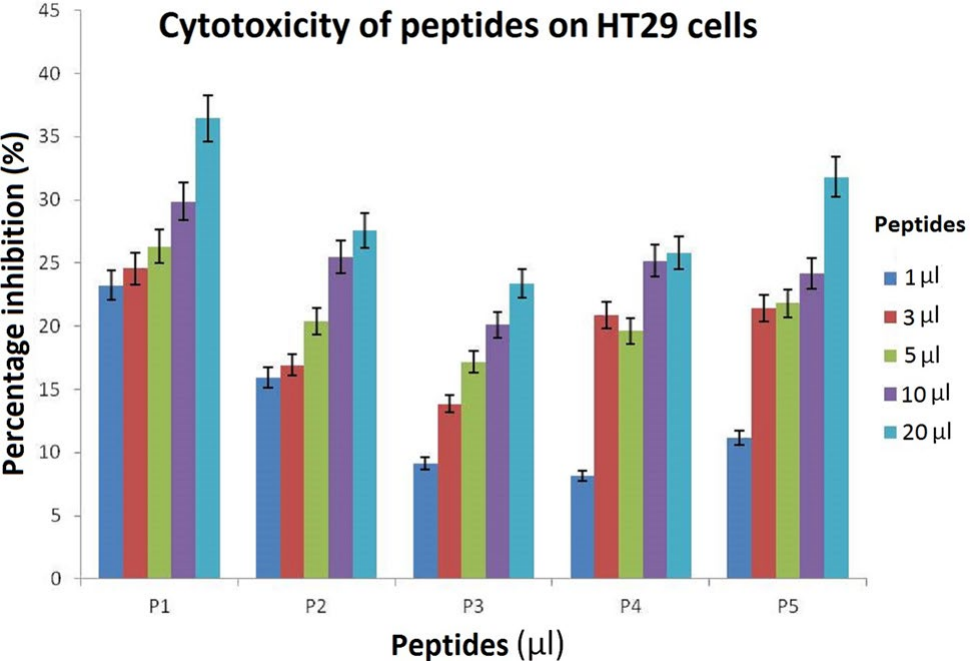
Graphical representation showing five hydrophobic peptides, P1, P2, P3, P4, and P5 screened for cytotoxic effects on HT-29 (cancerous) cell line determined by MTT assay. P1 showing the best IC50 value proving its efficacy

**Fig. 2:**
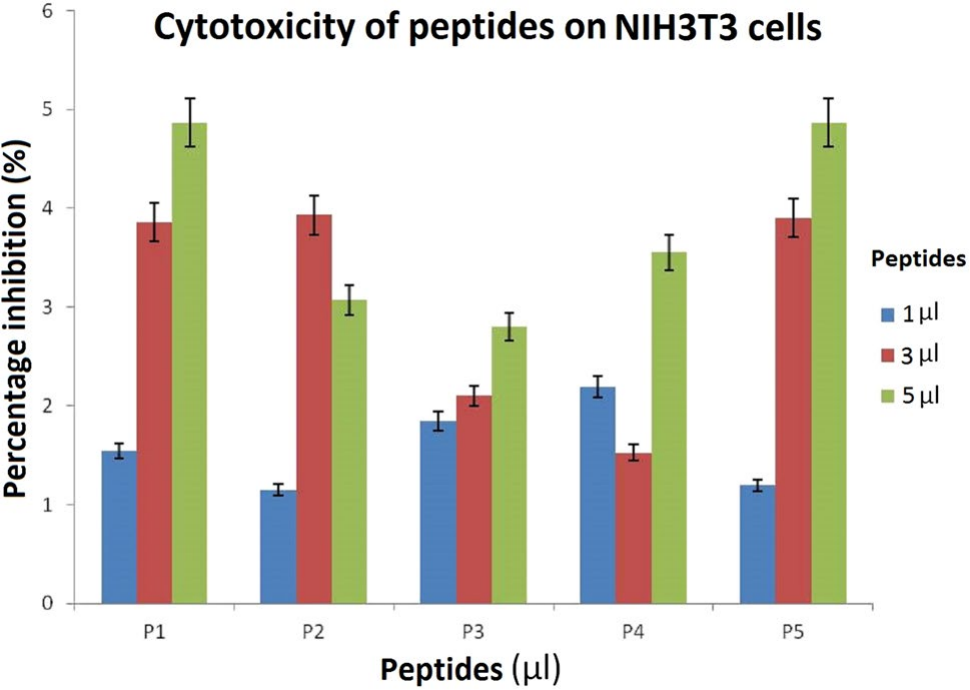
Cytotoxic effect of P1, P2, P3, P4, and P5 was determined on NIH3T3 (normal) cell lines by MTT assay as depicted in the graph

Among the five selected biologically important peptides, as low as 1 µg/mL concentration showed good results on the cancerous cells. Peptide 1 showed 23.22% inhibition, Peptide 3 showed 9.12% inhibition and Peptide 2 showed 15.93% inhibition at the lowest concentration (Fig. 1). At 5 µg/mL concentration, Peptides 4 and 5 showed inhibition around 20%. However, on NIH3T3 cells, all the peptides 1–5 n (Fig. 2). Because by comparison, the best results among the peptides on cancer cells were noticed only in case of peptide 1, we selected P1 for conjugation with the nanoparticles for further assay.

### Silver nano-peptide conjugation

**Fig. 3:**
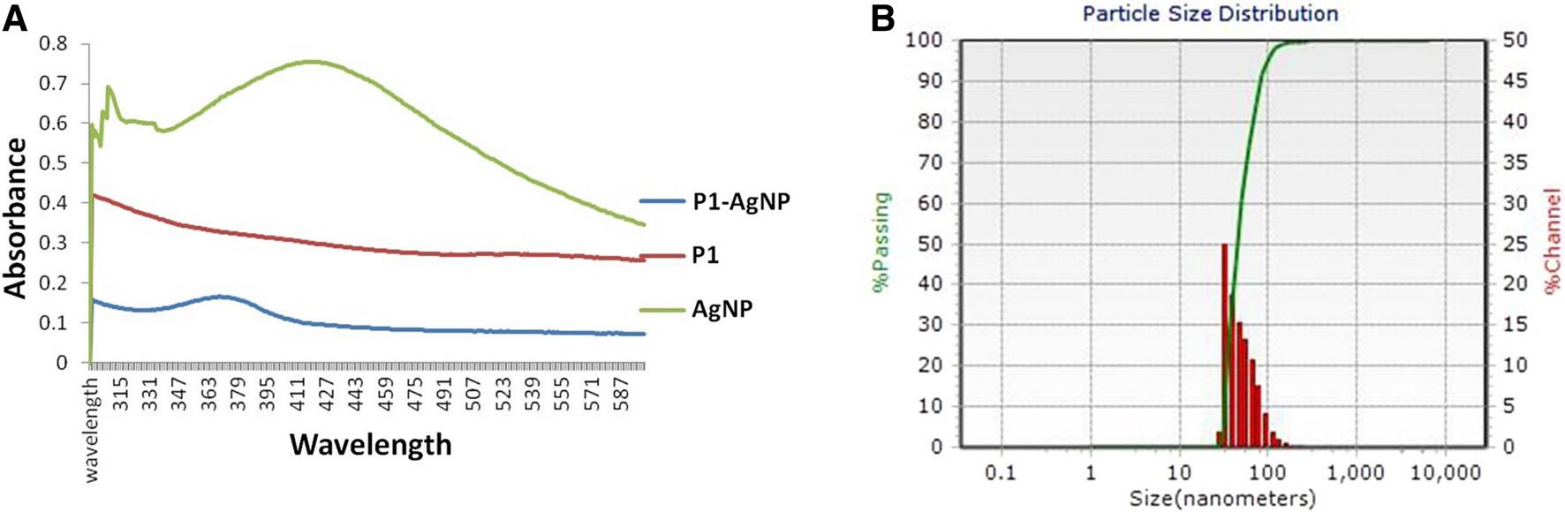
Characterization of the P1-AgNP conjugation by UV spectroscopy (**a**) and DLS (**b**) confirming the formation of the bio-conjugates

The silver nanoparticles–peptide conjugates were characterized and the analysis confirmed the occurrence of conjugation (Fig. 3). The UV spectroscopy peaks showed a shift from 421 to 373 nm due to the occurrence of conjugation of the silver nanoparticles to the peptides. Also, the DLS results invariantly showed an increase in the diameter of the particles from ~ 40 to 45.3 nm. These results were in recognition with the previous works (Akrami et al 2016).

### Gold–nano-peptide conjugation

**Fig. 4:**
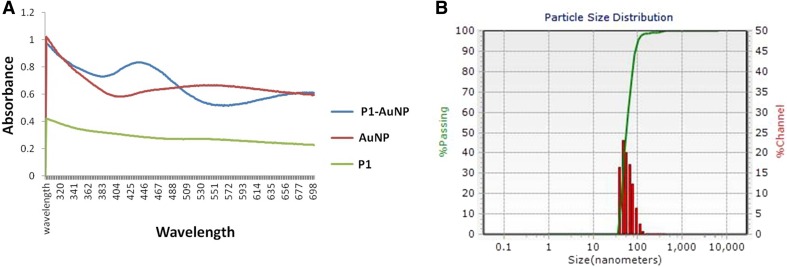
Characterization of the P1-AuNP conjugation by UV spectroscopy (**a**) and DLS (**b**) confirming the formation of the bio-conjugates

As described in the thesis previously, the average size of the particle synthesized by *A. fischeri* extracts was noted to be around 20–50 nm and was observed to be spherical in shape, monodispersive in nature. The peptide–NP conjugation was confirmed by UV peak shift from AuNP at 556 nm to P1–AuNP at 442 nm. Also, DLS results verified the peak shift with an increase in size of particles. AuNPs were around 50 nm; whereas conjugates were found to be in the range of 55 nm. Besides, the PDI of the particles was raised from 10.10 to 18.03 (Fig. 4). These conjugates were further used for assessing anticancer activity (Akrami et al 2016).

### Comparative assay on nanoparticles and their conjugates

### MTT assay

**Fig. 5:**
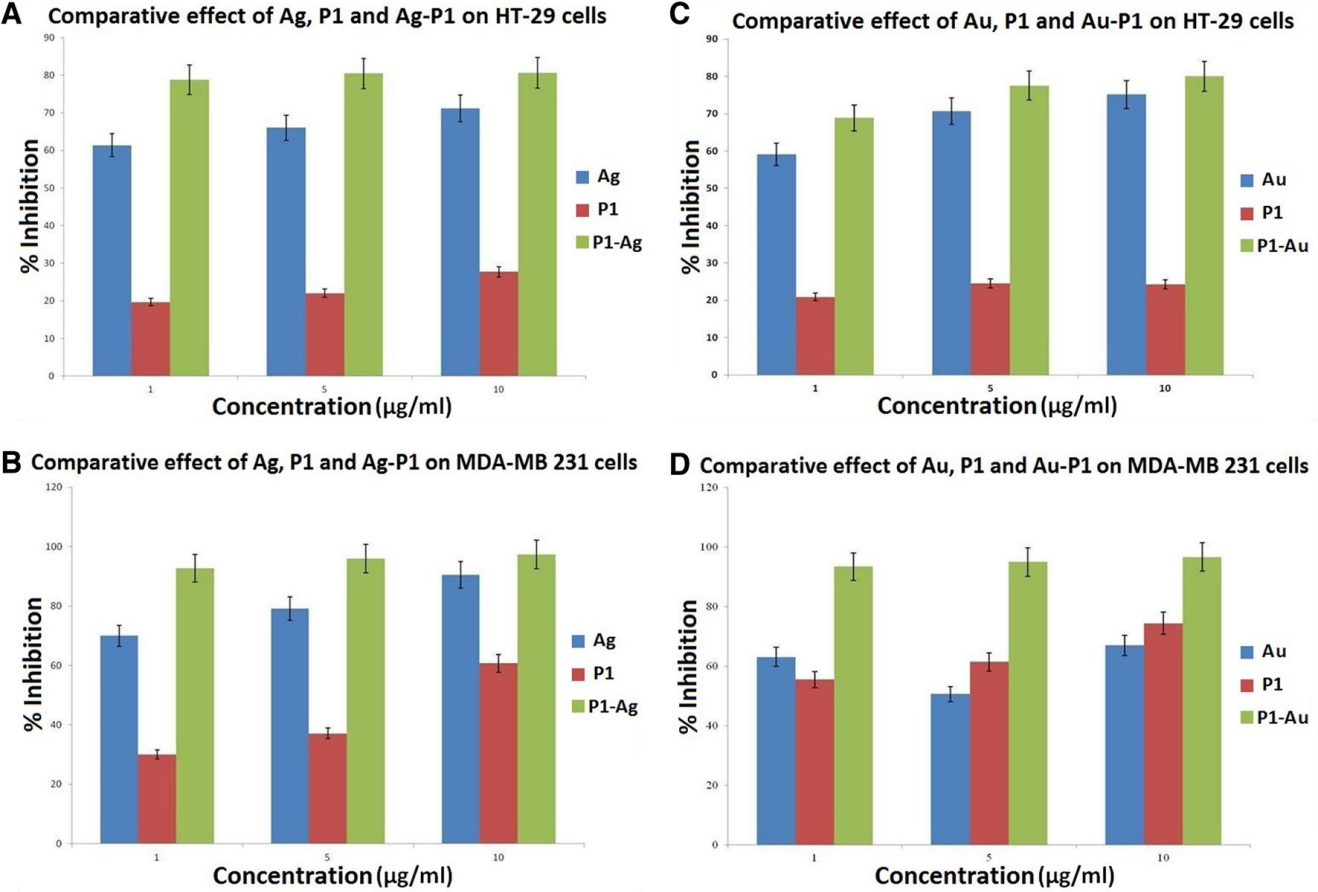
**a**–**d** Cytotoxicity of silver nanoparticles (AgNPs), gold nanoparticles (AuNPs), P1, P1-AgNP and P1-AuNP on HT-29 and MDA MB-231 according to MTT assay; Cytotoxicity measured using the MTT assay and plasma membrane integrity as endpoint of toxicity in colon cancer cell lines (HT 29) and breast cancer cell lines (MDA MB 231) following 24 h exposure to the nanoparticles and their conjugates. Cells exposed to concentration 0 received the maximal concentration of the dispersant and served as control

MTT assay was carried out as mentioned and the results were obtained. P1 showed inhibition around ~ 19%, AgNP showed inhibition around ~ 61% and P1–AgNP conjugate showed inhibition by 79% at a concentration of 1 µg/mL on HT-29 cells as depicted (Fig. 5a). However, when P1 demonstrated only around 29% and Ag around 70% of inhibition in case of breast cancer cells, Ag–P1 had an ability to kill cancer cells by up to ~ 93% (Fig. 5b).

Effectiveness of conjugating peptide with the bio-AuNPs was also depicted by the results obtained from MTT assay. P1 shows highest inhibition around 24%; AuNP shows a percentage inhibition only around 75% even at the highest concentration (10 µg/mL) for HT-29 colon cancer cells (Fig. 5c). The P1–AuNP conjugate shows an advantageous difference in percent inhibition when compared to P1 and AuNP singly. P1–AuNP shows inhibition of 68.86% at a concentration of as low as 1 µg/mL; whereas, neither P1 nor AuNP shows inhibition above conjugates at such low concentration. Similarly, in case of MDA MB-231 (breast cancer), the cell line was affected greatly by the P1–AuNP conjugates when compared to other two samples individually. In fact, P1–AuNP illustrated excellent inhibition of up to 96.66% on the breast cancer cell lines (Fig. 5d).

There can be several reasons behind the effects observed in this study. The large surface–volume ratio of the nanoparticles increases the peptide loading capacity mediated by cysteine linkers. On the other hand, stability of the particles is improved due to conjugation in avoiding unnecessary binding of serum proteins in the cell line media and, thus, becomes more effective against the cells. Also, the drug solubility augments and cellular uptake become easier. This advantage of the conjugation process can prove beneficial in the biomedical field to achieve drug target delivery, if the mechanism and path of the action of conjugates are studied elaborately.

To further conduct studies on these conjugates, fluorescent microscopy assays and DNA fragmentation analysis were carried out. These assays gave us better view of the possible events in inhibiting cancer cell growth by the conjugates.

### PI/AO staining for dead/live cells

The nucleic acid-binding dyes propidium iodide (PI) and acridine orange (AO) help us to detect the dead cells and live cells under the microscope. AO permeates both dead and live cells; thus, it stains all nucleated cells to generate green fluorescence, whereas PI identifies the dead cells only and fluoresce red. The treated cancer cells were both stained with PI and AO equally, and were observed under fluorescence microscope. The results are obtained on swapping of the images to have an apparent understanding of the drug effects (Hajrezaie et al 2015; Hajiaghaalipour et al 2015).

**Fig. 6:**
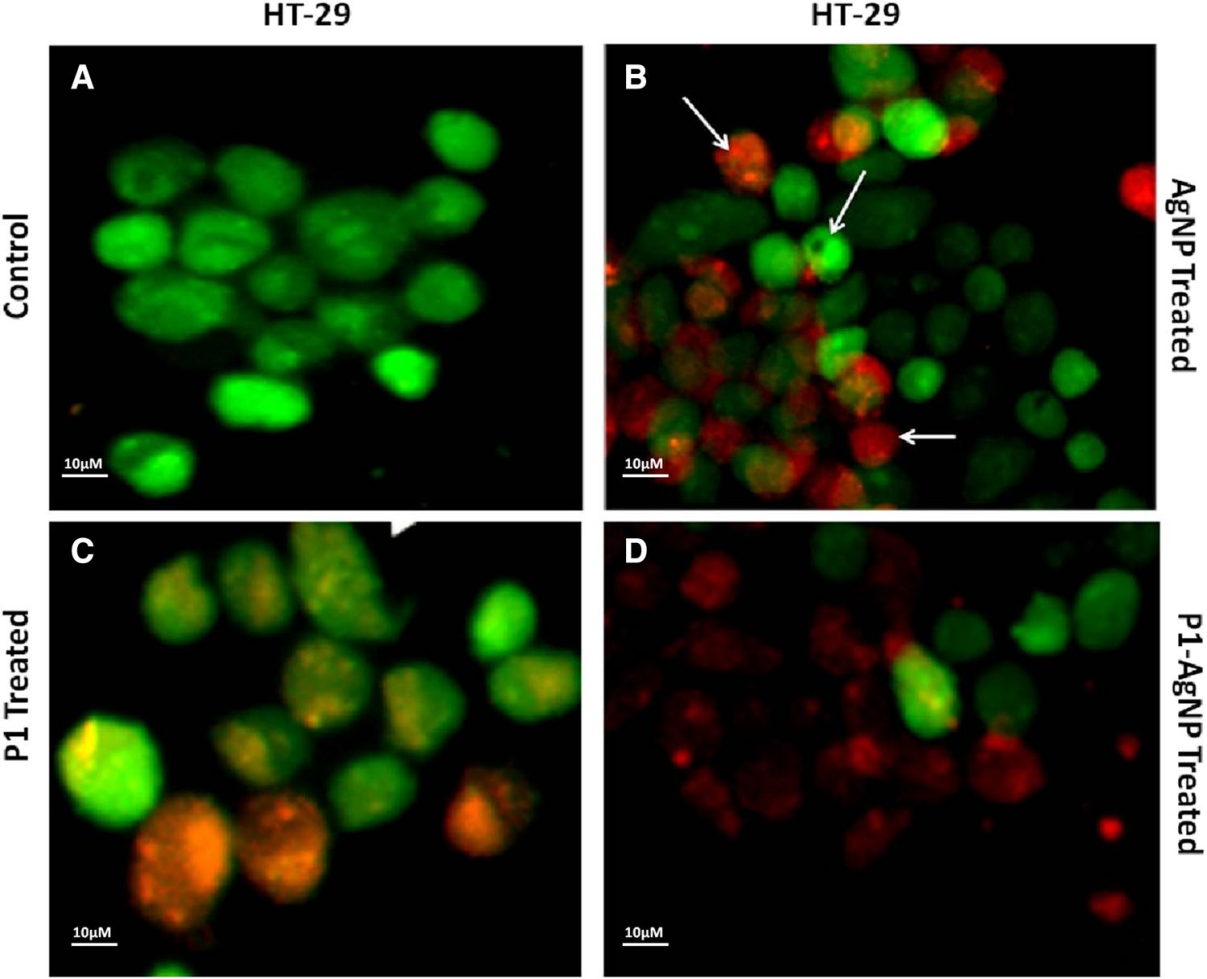
PI/AO fluorescent staining of HT-29 cells, (**a**) control and treated with (**b**) AgNP, (**c**) P1, (**d**) P1-AgNP. The cells when treated show apoptosis and deformation and turns orange and red in color

**Fig. 7:**
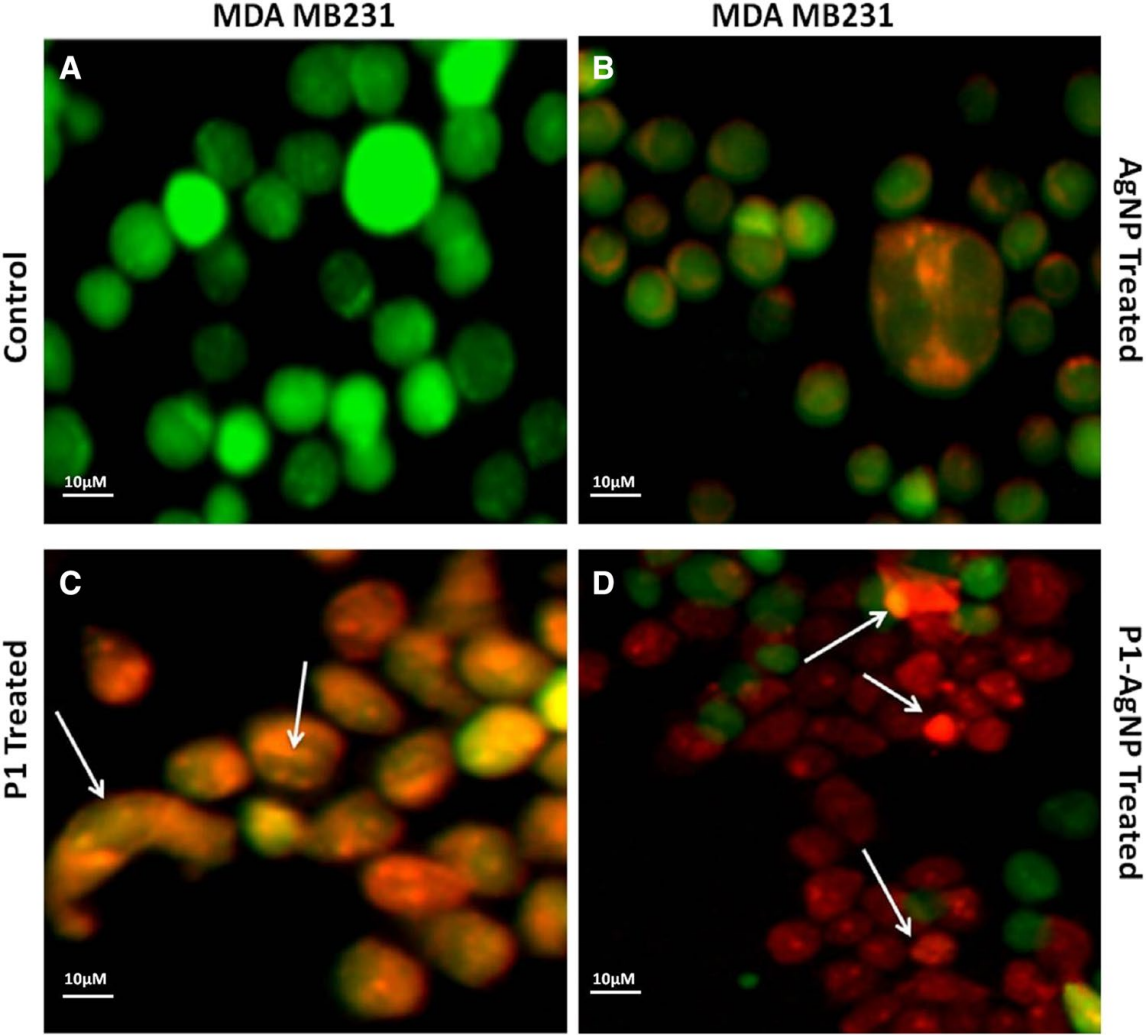
PI/AO fluorescent staining of MDA MB-231 cells, **a** control and treated with **b** AgNP, **c** P1, **d** P1-AgNP. Damage to the cell walls and structure was seen in the treated cells

**Fig. 8:**
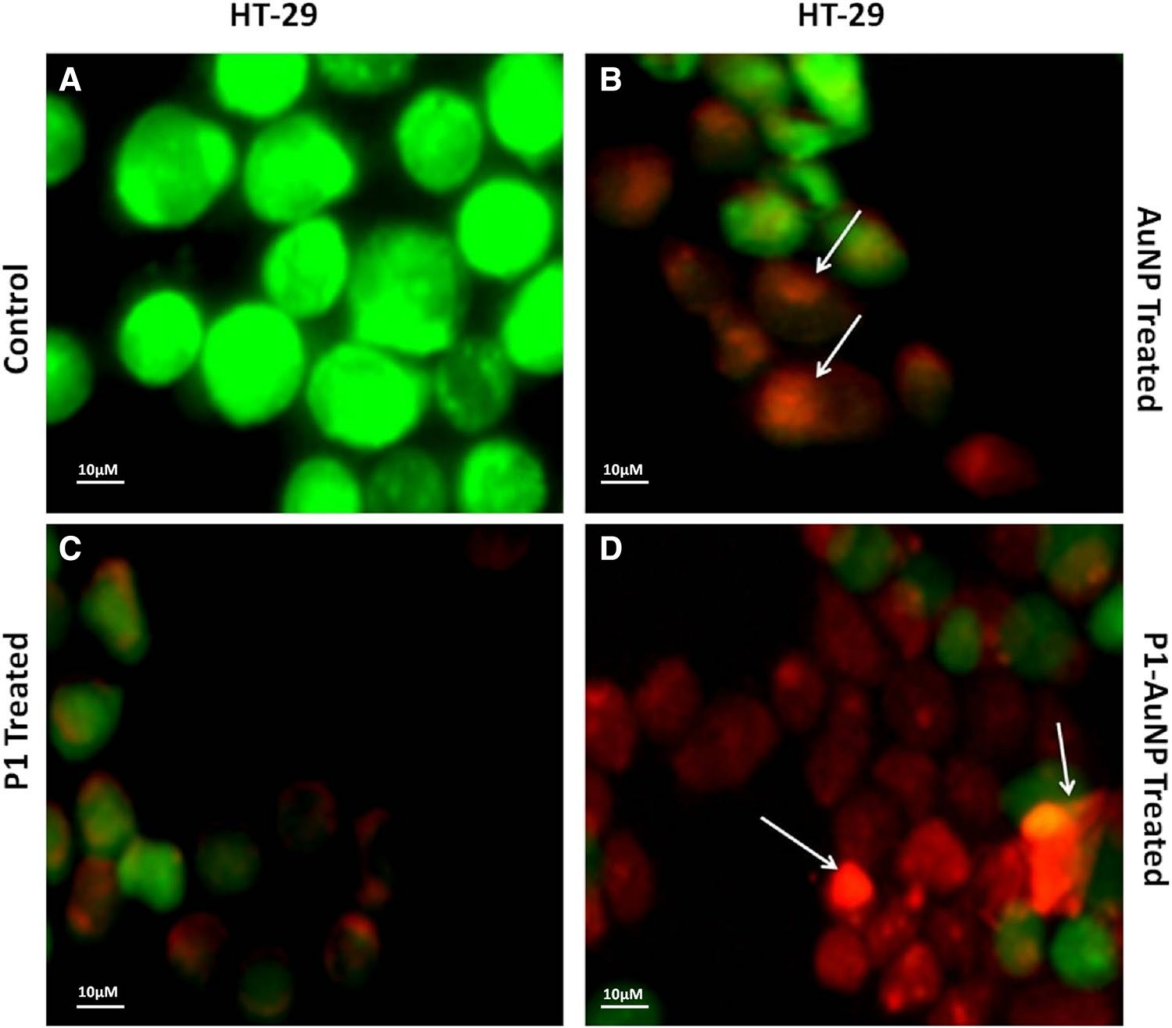
PI/AO fluorescent staining of HT-29 cells, **a** control and treated with **b** AuNP, **c** P1, **d** P1-AuNP. Conjugated sample showed the highest number of apoptotic cells

**Fig. 9:**
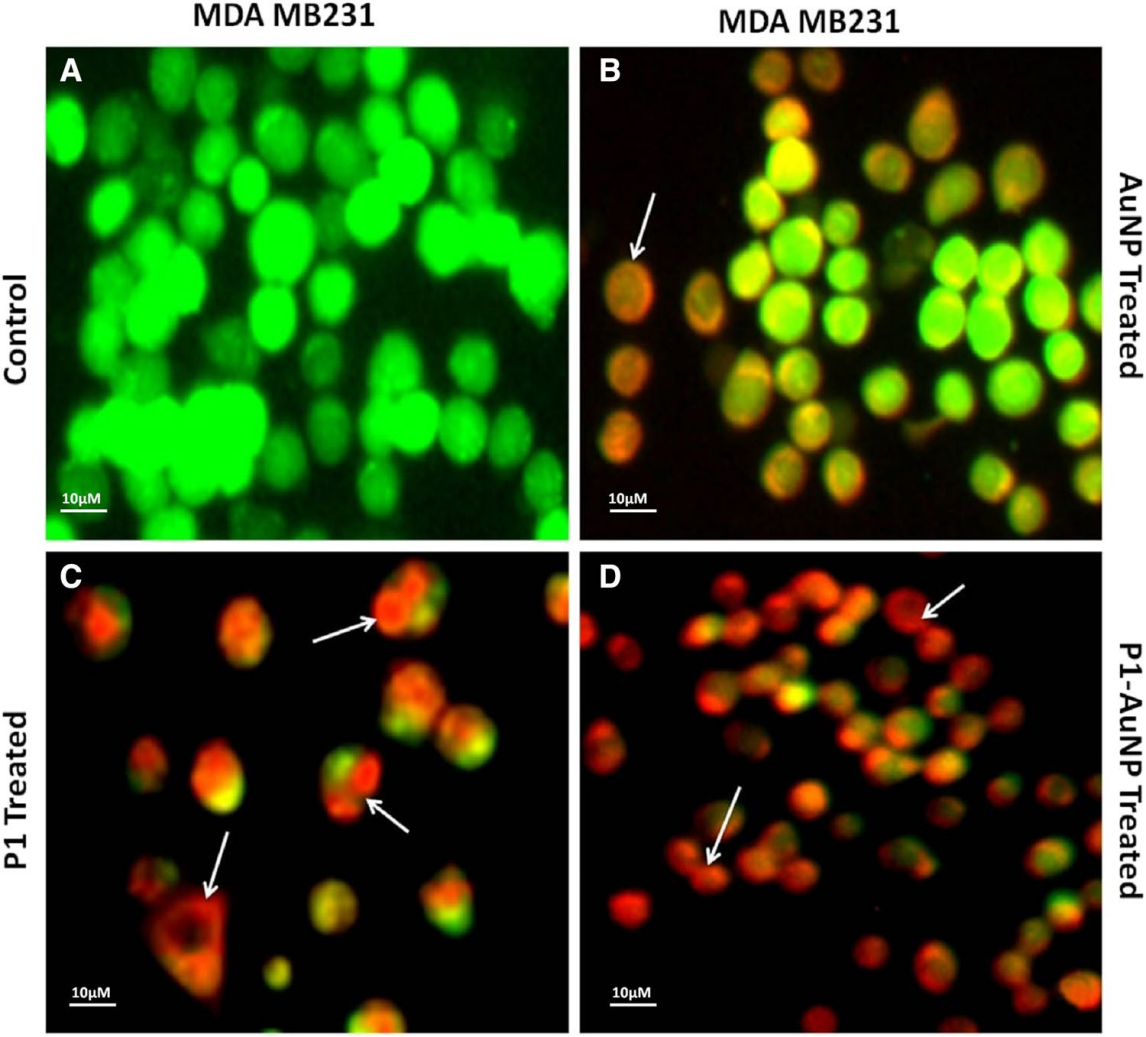
PI/AO fluorescent staining of MDA MB-231 cells, **a** control and treated with **b** AuNP, **c** P1, **d** P1-AuNP. Condensation of chromatin as well as deformation of the structure

Fluorescent images clearly explain the results and, thus, confirm the phenomenon behind cell death. Damages and swelling in treated cells are evident of apoptosis process when compared to the control. Images show cells treated with AuNPs consisting of cells dyed orange in color that are in the early apoptotic stage, which are very identical to the AgNP-treated cell images. The cells that were treated with only P1 also showed a number of apoptotic cells as well and very few dead cells dyed red in color. The cells, thus, treated with P1, AgNP and AuNP differ in shape from the control due to damage to the cell walls and structure. The cancer cells treated with the conjugated AuNP–P1 and AgNP–P1 samples as revealed clearly define the early apoptotic cells (orange) and dead cells (red). The cells, in fact, demonstrate condensation as well as deformation of the their structure which confirm the increased effect of the conjugated particles when compared to other images in addition to the control cells (Figs. 6, 7, 8, 9) (Hashim et al 2013, Saadat et al 2015).

### DAPI staining

**Fig. 10:**
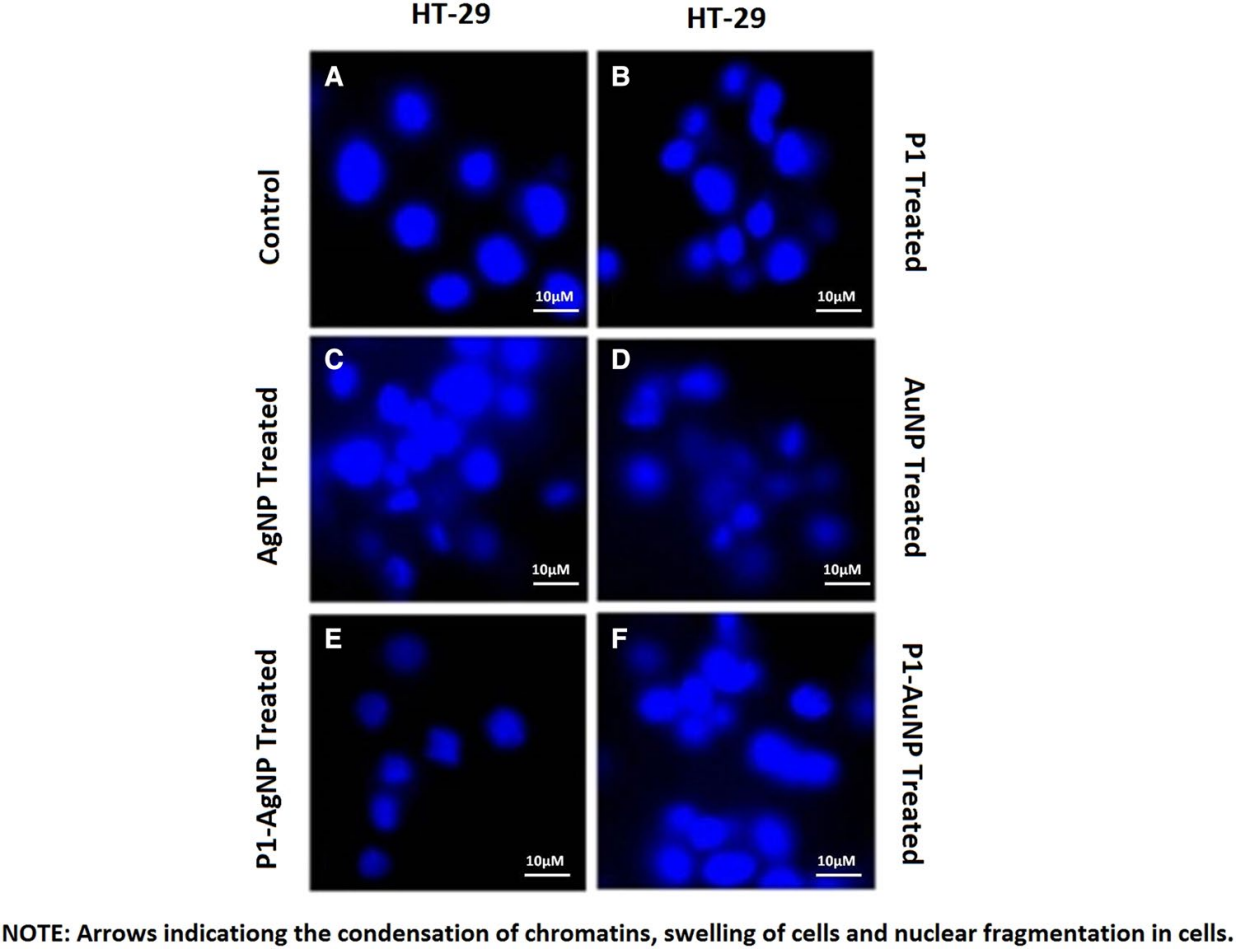
DAPI staining of HT-29, **a** control; **b** P1; **c** silver nanoparticles; **d** gold nanoparticles; **e** P1-AgNP; **f** P1-AuNP. Arrows indicating the condensation of chromatins, swelling of cells and nuclear fragmentation in cells

**Fig. 11:**
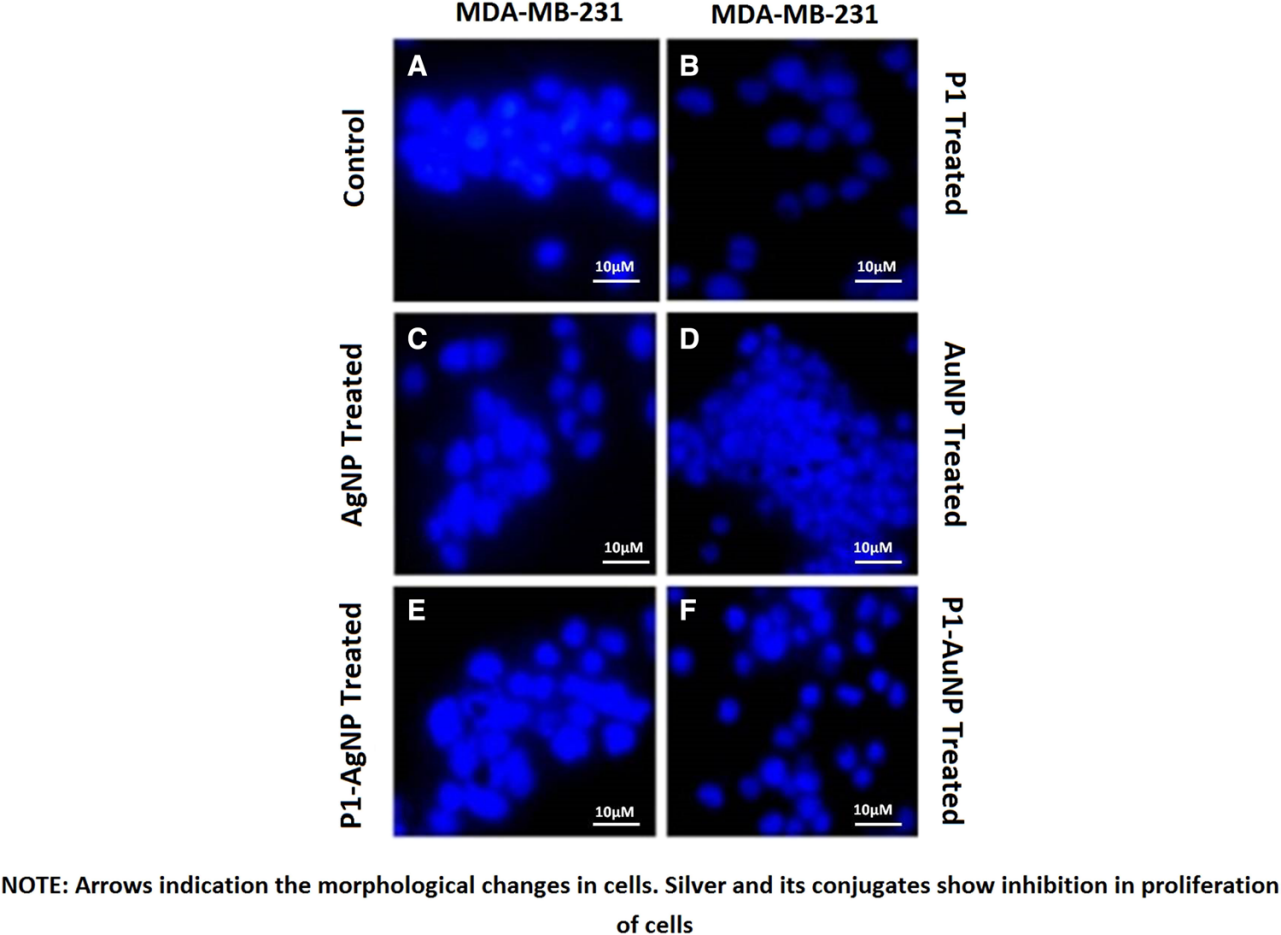
DAPI staining of MDA-MB-231, **a** control; **b** P1; **c** silver nanoparticles; **d** gold nanoparticles; **e** P1-AgNP; **f** P1-AuNP. Arrows indicate the morphological changes in cells. Silver and its conjugates show inhibition in proliferation of cells

The observation under fluorescent microscope explained the above results and confirmed the concept of cell death phenomenon. The images show damages and swelling in the treated cells. The morphological changes in the treated cells imply that the mode of action of nanoparticles and their conjugates caused apoptosis in the tumor cells. Nuclear fragmentation and condensation of chromatin were seen. Thus, the drugs used inhibit the proliferation of these tumor cells. Control helps us to compare the conditions of cells when stained before and after the treatment (Figs. 10, 11).

### DNA fragmentation assay

**Fig. 12:**
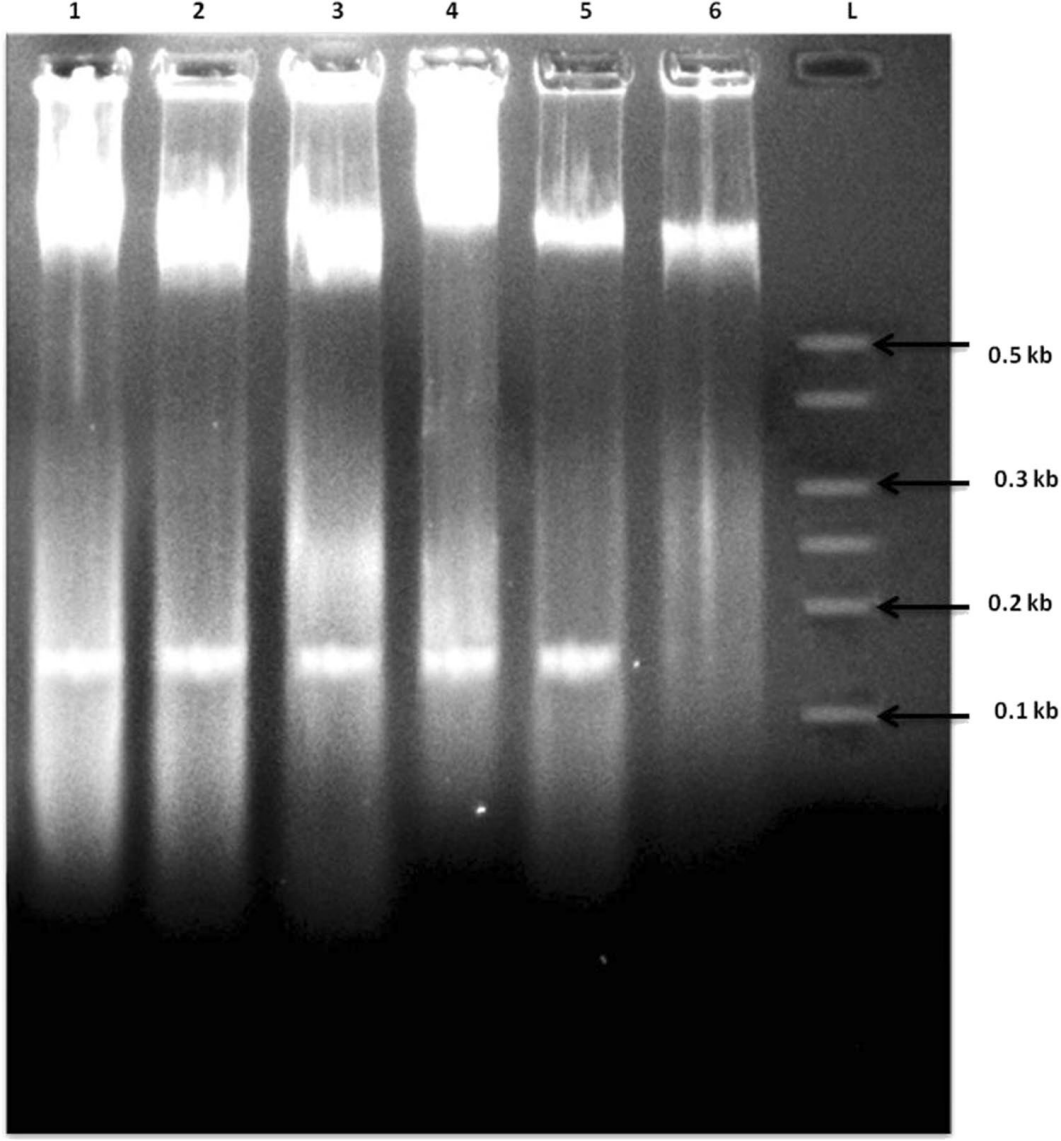
DNA fragmentation assay on HT-29 cells. Lane L: molecular marker, lane 6: control, lane 5: peptide 1 (P1), lane 4: AgNP, lane 3: AuNP, lane 2: P1-AgNP, lane 1: P1-AuNP

**Fig. 13:**
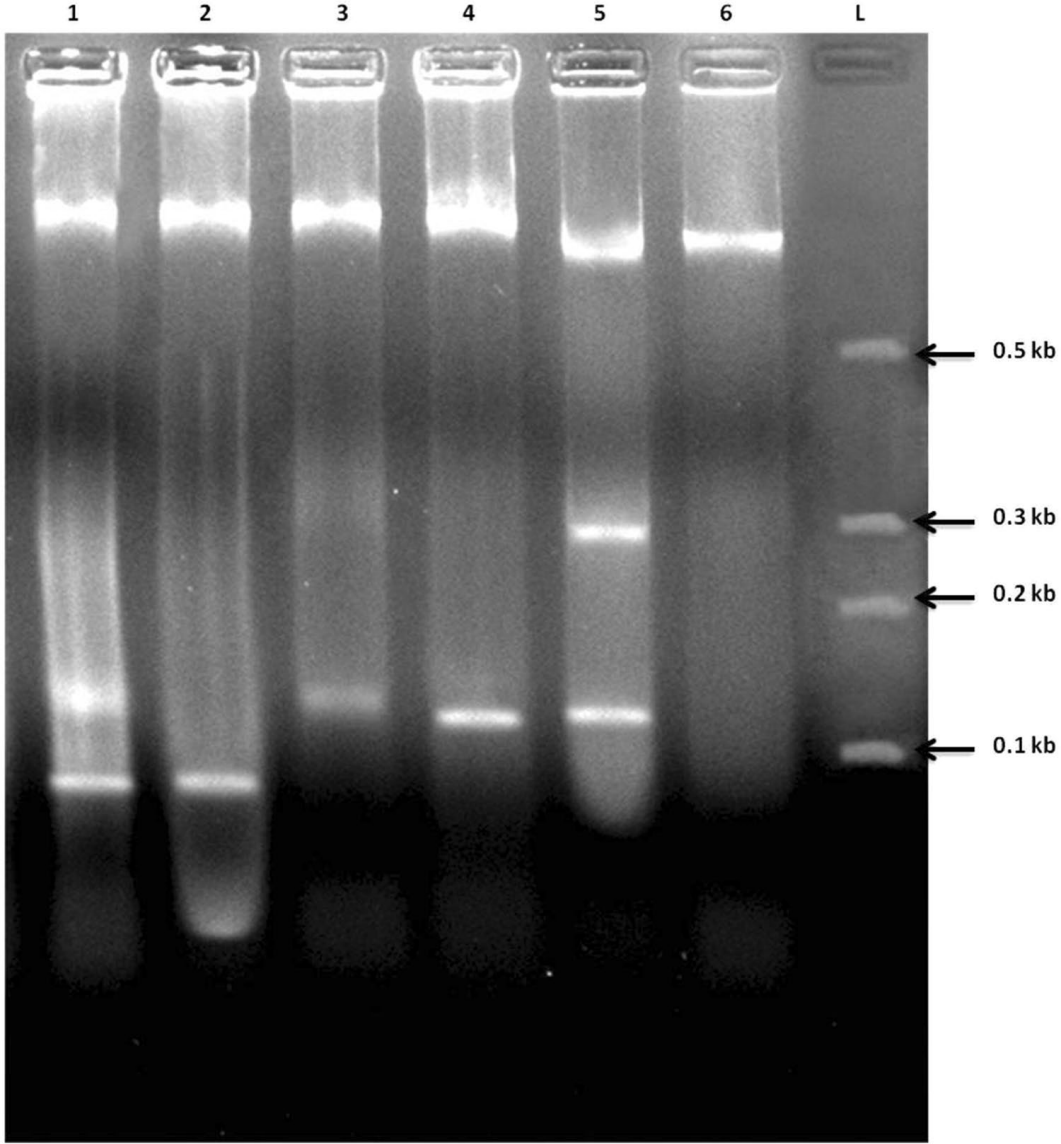
DNA fragmentation assay on MDA MB-231 cells. Lane L: molecular marker, lane 6: control, lane 5: peptide 1 (P1), lane 4: AgNP, lane 3: AuNP, lane 2: P1-AgNP, lane 1: P1-AuNP

As all three tested samples confirmed the death of cancer cells in previous assays, DNA fragmentation patterns were obtained to authenticate the cause of cell death to be either necrosis or apoptosis. The apoptosis can be visualized as a ladder pattern of 100–125 bp due to DNA cleavage which had been activated by a nuclear endonuclease observed on the standard agarose gel electrophoresis. The fragmented smear was observed as an evidence of clear elevated DNA degradation in HT-29 and MDA MB-231 cancer cells in case of AgNP–P1 and AuNP–P1 conjugate samples when compared to nanoparticles and peptide individually (Figs. 12, 13) (Balaji and Gothandam 2016, Saadat et al 2015, Teerasripreecha et al 2012).

### Statistical analysis

The two-way analysis of variance (ANNOVA) showed *F* value more than F critical for all the collected data analysis. The statistical analysis proved *p* < 0.05; thus, null hypothesis can be neglected for each comparative effect determined by MTT assay and from derived data. Also, Table 1 depicts the calculated IC50 of nanoparticles and their conjugates on the cancer cell lines.

**Table 1:** IC50 (µg/mL) values calculated from the different cytotoxicity assay systems in the colon cancer (HT 29) cell lines and breast cancer (MDA MB 231) cell lines after exposure to silver nanoparticles (AgNPs), gold nanoparticles (AuNPs), peptide (P1), P1-AgNP and P1-AuNP

Concentration (µg/mL)	% inhibition									
	HT 29					MDA MB 231				
	Ag	Au	P1	Ag-P1	Au-P1	Ag	Au	P1	Ag-P1	Au-P1
1	61.38	59.11	19.69	78.85	68.86	70	63.10	29.98	92.79	93.40
5	66.03	70.74	22.05	80.48	77.54	79.16	50.66	37.11	96.04	94.97
10	71.21	75.16	27.72	80.63	80.06	90.50	66.95	60.69	97.34	96.66
IC50 (µg/mL)	7.83	7.68	4.43	8.88	8.29	6.66	0.66	6.25	4.24	2.00

## Conclusion

*Aspergillus fischeri*-mediated biosynthesized silver and gold nanoparticles were focused upon in this study due to their small size and unique superior effect on colon cancer cells as well as breast cancer cells at an extremely low concentration of 1 µg/mL. To aim more precise accumulation of nanoparticles in various tumors than in other normal tissues, targeted delivery has become one of the most challenging aspects in nano-biotechnology in recent days. Hence, our research thoroughly deals with systematically developed small molecules that will have important applications in the treatment of malignant tumors and eventually other diseases that are dependent on nanotechnology. Here, we confirm the augmented effect of peptide–AgNPs and peptide–AuNPs conjugates on colon and breast cancer cells. Also, we verify apoptosis or DNA damage being the causes behind the inhibitory effect of these conjugates. We compare the outcome with effects of AgNP, AuNP and P1 individually and find out that in this case, cell death or apoptosis occurs slower in rate. This can be explained by specific targeted delivery of the drugs that damage the DNA in advanced pace. Further, the designed synthesis of the peptide–nanoparticle conjugates and determining the mechanisms behind elevated necrosis or apoptosis in cancer cells can help in the improved delivery of compounds and provide developed imaging and therapeutic options.

## References

[CR1] Akrami M, Balalaie S, Hosseinkhani S, Alipour M, Salehi F, Bahador A, Haririan I (2016). Tuning the anticancer activity of a novel pro-apoptotic peptide using gold nanoparticle platforms. Sci Rep.

[CR2] Balaji K, Gothandam KM (2016). Cytotoxic effect on cancerous cell lines by biologically synthesized silver nanoparticles. Braz Arch Biol Technol.

[CR3] Banerjee K, Ravishankar VR (2017). *Aspergillus fischeri* mediated biosynthesis of gold nanoparticles and their beneficially comparative effect on normal and cancer cell lines. Pharma Nanotechnol.

[CR4] Gindy ME, Prud’homme RK (2009). Multifunctional nanoparticles for imaging, delivery and targeting in cancer therapy. Expert Opin Drug Deliv.

[CR5] Hajiaghaalipour F, Kanthimathi MS, Sanusi J, Rajarajeswaran J (2015). White tea (*Camellia sinensis*) inhibits proliferation of the colon cancer cell line, HT-29, activates caspases and protects DNA of normal cells against oxidative damage. Food Chem.

[CR6] Hajrezaie M, Paydar M, Looi CY, Moghadamtousi SZ, Hassandarvish P, Salga MS, Karimian H, Shams K, Zahedifard M, Majid NA, Ali HM, Abdulla MA (2015). Apoptotic effect of novel Schiff based CdCl_2_ (C_14_H_21_N_3_O_2_) complex is mediated via activation of the mitochondrial pathway in colon cancer cells. Sci Rep.

[CR7] Hambley TW, Hait WN (2009). Is anticancer drug development heading in the right direction?. Can Res.

[CR8] Hashim FJ, Shawkat MS, Aljewari H (2013). Anti-cancer effect of curcuma longa on leukemic cell lines evaluated by apoptosis and comet assay. Int J Pharm Pharma Sci.

[CR9] Heath JR, Davis ME (2008). Nanotechnology and Cancer. Ann Rev Med.

[CR10] Inkielewicz-Stepniak I, Santos-Martinez MJ, Medina C, Radomski MW (2014). Pharmacological and toxicological effects of co-exposure of human gingival fibroblasts to silver nanoparticles and sodium fluoride. Int J Nanomed.

[CR11] Rai R, Raghothama S, Sridharan R, Balaram P (2006). Tuning the b-turn segment in designed peptide b-hairpins: construction of a stable type I0 b-turn nucleus and hairpin-helix transition promoting segments. Pept Sci.

[CR12] Ruoslahti E, Bhatia SN, Sailor MJ (2017). Targeting of drugs and nanoparticles to tumors. J Cell Bio.

[CR13] Saadat YR, Saeidi N, Vahed SZ, Barzegari A, Barar J (2015). An update to DNA ladder assay for apoptosis detection. BioImpacts.

[CR14] Shrivastava S, Bera T, Roy A, Singh G, Ramachandrarao P, Dash D (2007). Characterization of enhanced antibacterial effects of novel silver nanoparticles. Nanotechnology.

[CR15] Teerasripreecha D, Phuwapraisirisan P, Puthong S, Kimura K, Okuyama M, Mori H, Kimura A, Chanchao C (2012). In vitro antiproliferative/cytotoxic activity on cancer cell lines of a cardanol and a cardol enriched from Thai Apis mellifera propolis. BMC Comp Alter Me.

